# Compositional and mechanical properties of growing cortical bone tissue: a study of the human fibula

**DOI:** 10.1038/s41598-019-54016-1

**Published:** 2019-11-26

**Authors:** Emmanuelle Lefèvre, Delphine Farlay, Yohann Bala, Fabien Subtil, Uwe Wolfram, Sébastien Rizzo, Cécile Baron, Philippe Zysset, Martine Pithioux, Hélène Follet

**Affiliations:** 1 Aix-Marseille Univ., CNRS, ISM Inst Movement Sci, Marseille, France; 2 Department of Orthopaedics and Traumatology, Institute for Locomotion, APHM, Sainte-Marguerite Hospital, Marseille, France; 3 Univ Lyon, Université Claude Bernard Lyon 1, INSERM, Lyos UMR1033, F69622 Lyon, France; 4 Laboratoire Vibrations Acoustique, INSA Lyon, Campus LyonTech la Doua, F69621 Villeurbanne Cedex, France; 5 Univ Lyon, Université Claude Bernard Lyon 1, Equipe Biostatistique Santé – LBBE, F69003 Lyon, France; 6 School of Engineering and Physical Science, Heriot-Watt University, Edinburgh, United Kingdom; 7 ARTORG Center for biomedical engineering research, University of Bern, Bern, Switzerland

**Keywords:** Ageing, Bone, Bone quality and biomechanics

## Abstract

Human cortical bone contains two types of tissue: osteonal and interstitial tissue. Growing bone is not well-known in terms of its intrinsic material properties. To date, distinctions between the mechanical properties of osteonal and interstitial regions have not been investigated in juvenile bone and compared to adult bone in a combined dataset. In this work, cortical bone samples obtained from fibulae of 13 juveniles patients (4 to 18 years old) during corrective surgery and from 17 adult donors (50 to 95 years old) were analyzed. Microindentation was used to assess the mechanical properties of the extracellular matrix, quantitative microradiography was used to measure the degree of bone mineralization (DMB), and Fourier transform infrared microspectroscopy was used to evaluate the physicochemical modifications of bone composition (organic versus mineral matrix). Juvenile and adult osteonal and interstitial regions were analyzed for DMB, crystallinity, mineral to organic matrix ratio, mineral maturity, collagen maturity, carbonation, indentation modulus, indicators of yield strain and tissue ductility using a mixed model. We found that the intrinsic properties of the juvenile bone were not all inferior to those of the adult bone. Mechanical properties were also differently explained in juvenile and adult groups. The study shows that different intrinsic properties should be used in case of juvenile bone investigation.

## Introduction

From a clinical point of view, juvenile bone is of interest since various congenital, acquired diseases or trauma influence bone development in childhood and adolescence. Juvenile bone growth is well described^1^ and starts with the formation of primary bone, formed by the early primary Haversian system, which is then remodeled into a more complex secondary Haversian system with new lamellar bone and oriented cylindrical osteons. Bone modeling allows increasing bone size, as resorption and formation occur simultaneously on different surfaces of the bone (periosteal apposition and endocortical resorption). After reaching final size, bone remodeling occurs with the coupling of resorption and formation at the same location. This could lead to different intrinsic properties. Even if this growth process is well described, tissue mechanical properties and their relationships with compositional properties are not well established in comparison to adult bone.

Cortical bone contains both osteonal and interstitial tissue. Osteons are comprised of 5 to 30 concentric lamellae with different collagen fibril orientations^2–5^ that are arranged around Haversian canals, which ensure an adequate blood supply and innervation of bone^6^. Secondary osteons are the product of bone remodeling in which old bone is replaced with new bone^7^. Interstitial tissue is found between osteons and is made of osteonal remnants that remain after bone remodeling. Both osteons and interstitial bone represent bone structural units (BSUs). The characterization of juvenile bone *in vivo* using HRpQCT demonstrated that the transient increase in distal forearm fractures during adolescent growth is associated with alterations in cortical bone which include cortical thinning and increasing porosity^8^. The assessment of mineral metabolism is also complex in pediatrics^9^. Characterization of juvenile bone on iliac biopsies by histomorphometry^10,11^ from 58 healthy subjects showed that the ilium growth occurred through simultaneous periosteal and endosteal resorption and apposition in inner and outer cortices leading to an increase of cortical width from 0.52 mm at an age of 2 years to 1.14 mm by the age of 20 years. It also showed that a lateral modeling drift of the inner cortex encroaches on the marrow cavity. According to Bala *et al*., the pore volume fraction did not significantly differ between children and adults but originates from different microarchitectural patterns^12^.

The mechanical properties of bone tissue change during growth. For example, bone becomes stiffer and more resistant to fracture^13–15^. As bone is a hierarchical bio-composite material made of an organic matrix (a network of type I collagen fibrils) filled and impregnated with a mineral component consisting of apatite crystals that interact with collagen fibrils^16^, mineral content plays a major role in bone strength^17,18^. However, there are very few data on characterization of healthy juvenile bone at the tissue level (at the micro meters length-scale)^19,20^ and none contrasting such data with a conjoined adult set of samples. The micromechanical properties of bone can be assessed by microindentation tests which is not available for juvenile bone. In adult bone, plane strain modulus was in the range of 7 to 35 GPa^21–25^ and cortical bone stiffness is predominantly associated with mineral content and bone density while cortical bone toughness correlates with the quality of the collagen matrix^26^. At the BSU level, axial elastic properties and hardness of bone are dependent on the degree of mineralization^6,27^. Using quantitative backscattered electron imaging in juvenile iliac crest bone biopsies, Fratzl-Zelman *et al*. showed that at the trabecular level, no variation of bone mineralization with age was present. However, the average values of mineralization density were slightly lower than in the adult reference population. The cortices appeared to be less mineralized than the trabecular bone^10,19^ with a lower mineralization in the inner compared to the outer cortex between 1.5 year to 14 years. Mechanical properties of cortical bone also depend on the size and distribution of mineral crystals^28,29^. Characteristics such as crystallinity, i.e. crystal size and/or lattice perfection, may influence bone mechanical properties^30,31^. The role of collagen, however, remains unclear^32^ but cortical bone toughness is reduced by dehydration^33,34^ and crack bridging is a predominant mediator of cortical bone toughening^35,36^. The elastic properties of lamellae depend on the orientation of collagen fibers^37–39^ and it has been demonstrated that, at the lamellar level, collagen is involved in plastic properties^6,36^. As described by Bala *et al*., mineral density (degree of mineralization of bone, DMB), mineral quality (crystallinity), and collagen maturity (age of collagen matrix) are the minimum necessary variables required to define intrinsic properties of adult bone tissue. Thus, they are likely to be the predictive indicators of bone mechanical properties at the osteon level^6^. To date, this is not clear in case of the mechanical properties of juvenile bone, especially when differentiating between osteonal and interstitial regions and a significant gap of knowledge concerning juvenile bone in comparison to adult tissue exists.

Therefore, the aim of this study was for the first time to analysis together the composition and indentation properties of osteonal and interstitial tissue from juvenile and adult bone to understand how structure, composition, and mechanics affect each other. For this purpose, micromechanical properties will be assessed at the same location by instrumented microindentation^40^, the degree of mineralization by digitized microradiography^41^, and mineral and organic characteristics (crystallinity, mineral/matrix ratio, mineral and collagen maturities, carbonate content) by Fourier Transform Infrared Microspectroscopy (FTIRM)^42–44^. Specifically, we investigated (i) the relation between tissue mechanical as well as compositional properties with donor age, (ii) the relation of these properties with tissue age, and (iii) their interrelation with each other. We hypothesized that intrinsic properties would change with age in juveniles but not in adults.

## Results

We analyzed the effect of gender using a multiple regression on adult and juvenile bone, and no influence of sex was found.

### Evolution of parameters with chronological age in juveniles and adults (osteonal and interstitial tissue properties)

**Figure 1:**
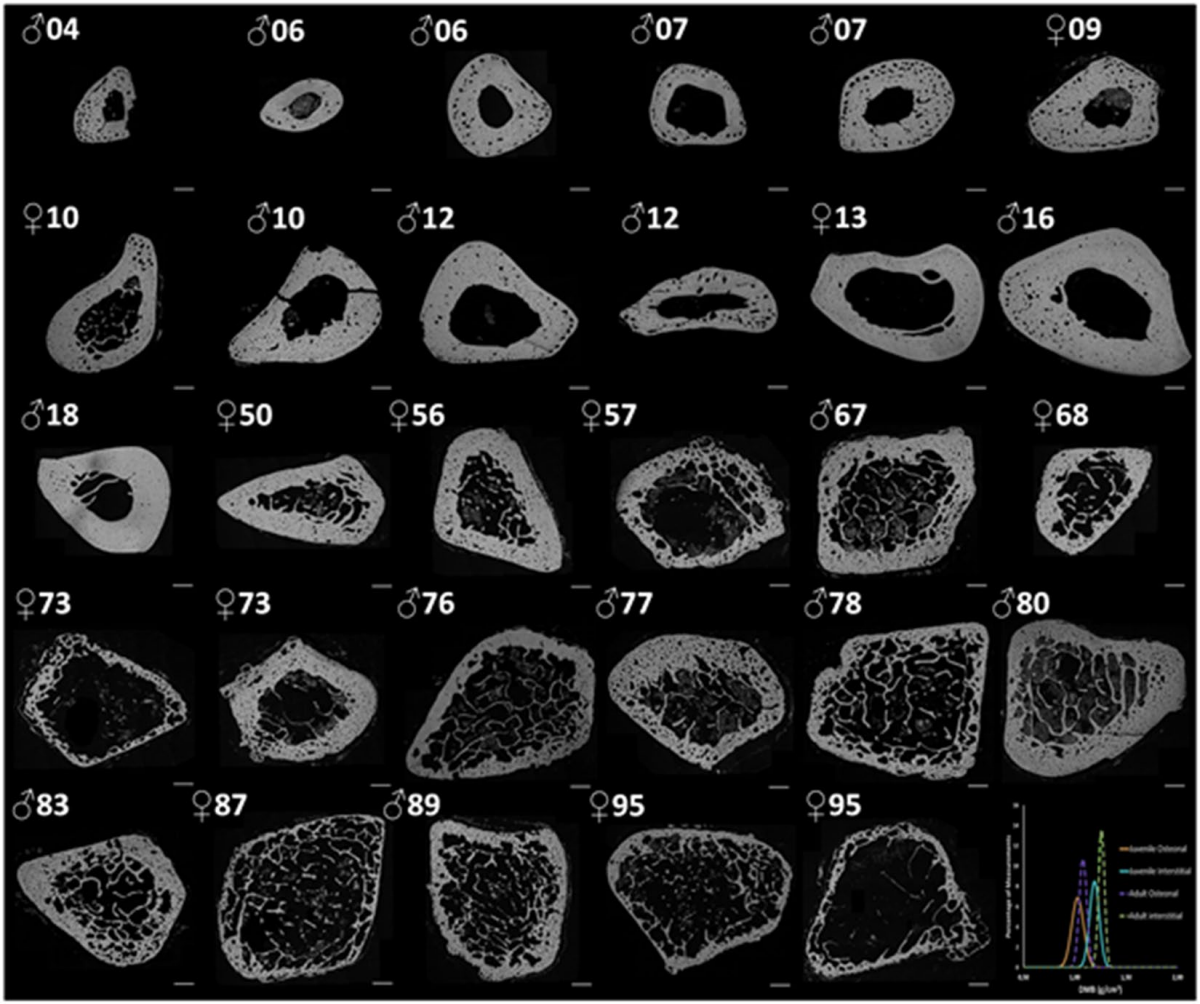
Top left to bottom right: Evolution of transverse section of fibula with age (age and sex are indicated above each section), taken at the same location. Illustrations come from DMB measurement by X-rays. Note the evolution of the cortex and trabeculae. Scale bars represent 1.5 mm. The right bottom graph shows the DMB distribution in juveniles (Solid line) and adults (Dot line) in osteonal and interstitial bone.

**Figure 2:**
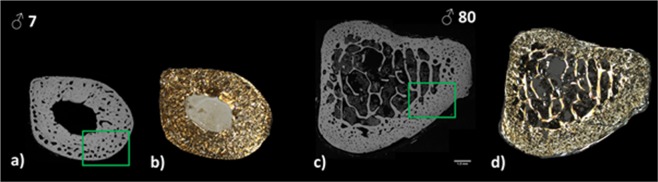
Transverse fibulae section at low magnification at two ages (x1). (**a–c)** Quantitative digitized microradiography was used to measure the degree of mineralization of bone (DMB, g/cm^3^), and (**b–d**) identical sections observed in polarized light. Rectangles show the location of the high magnification illustrations in Figs. 3 and 4. Illustrations will be available at high resolution.

**Figure 3:**
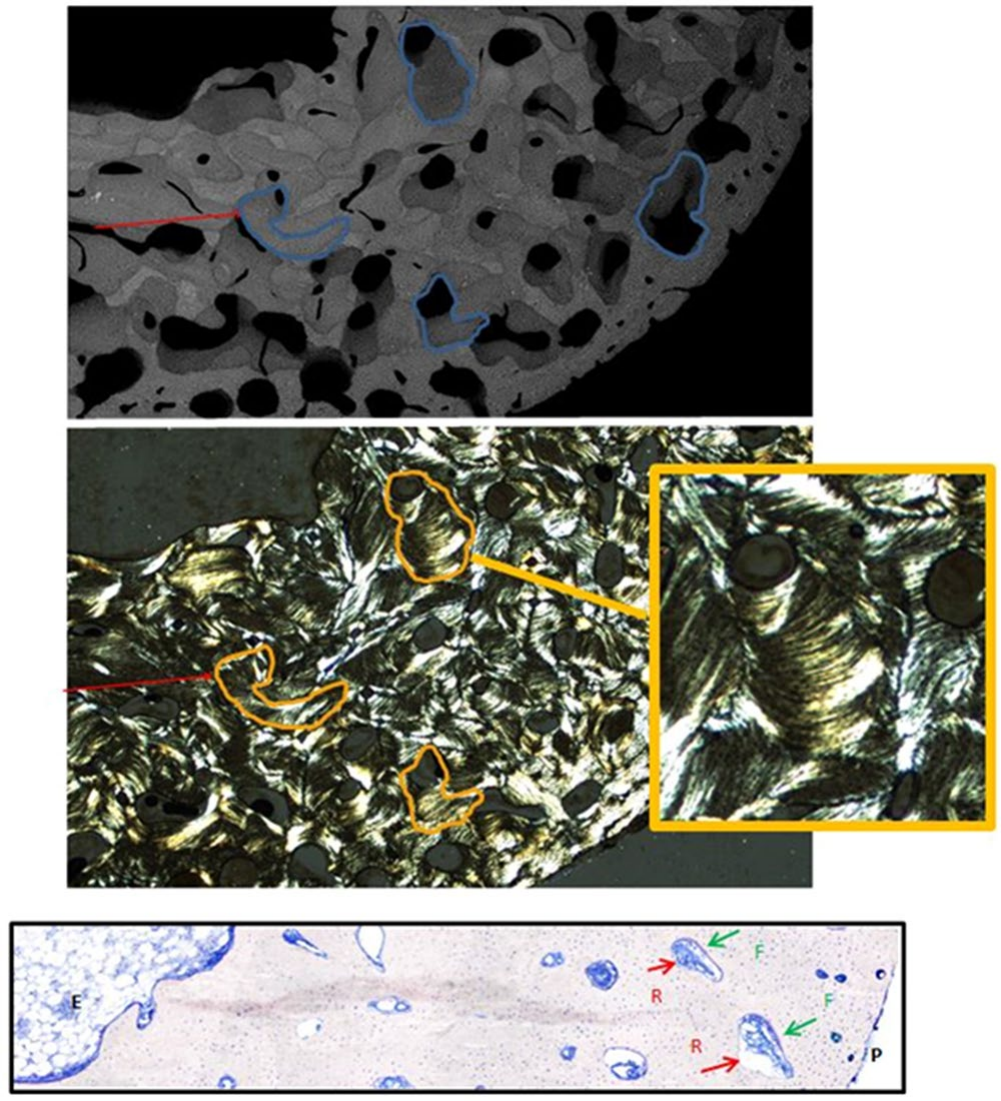
At high magnification (x2.5) from left Fig. 2, top image shows the drifting osteons present in juvenile bone. Note the heterogeneity of the mineralization, with dark (low mineralization) and white osteons (higher mineralization). Red arrow and blue lines point of drifted osteons as shown in Robling *et al*. 1999. Middle image shows the corresponding section seen in polarized light. Drifting osteons exhibit a variation in the direction of transverse drift along their longitudinal axes, intermitent regions of concentric morphology and change in drift direction over time. Bottom image illustrates at 8 µm thin-section of May-Grünwald Giemsa staining, from endosteal (E) to periosteal (P) area, at high magnification (x10). Bone modeling is characterized by a formation (F) and resorption (R) at different location. Formation is mostly oriented throught the perioste and resorption in endoste, allowing the bone growth. Illustrations will be available at high resolution

**Figure 4:**
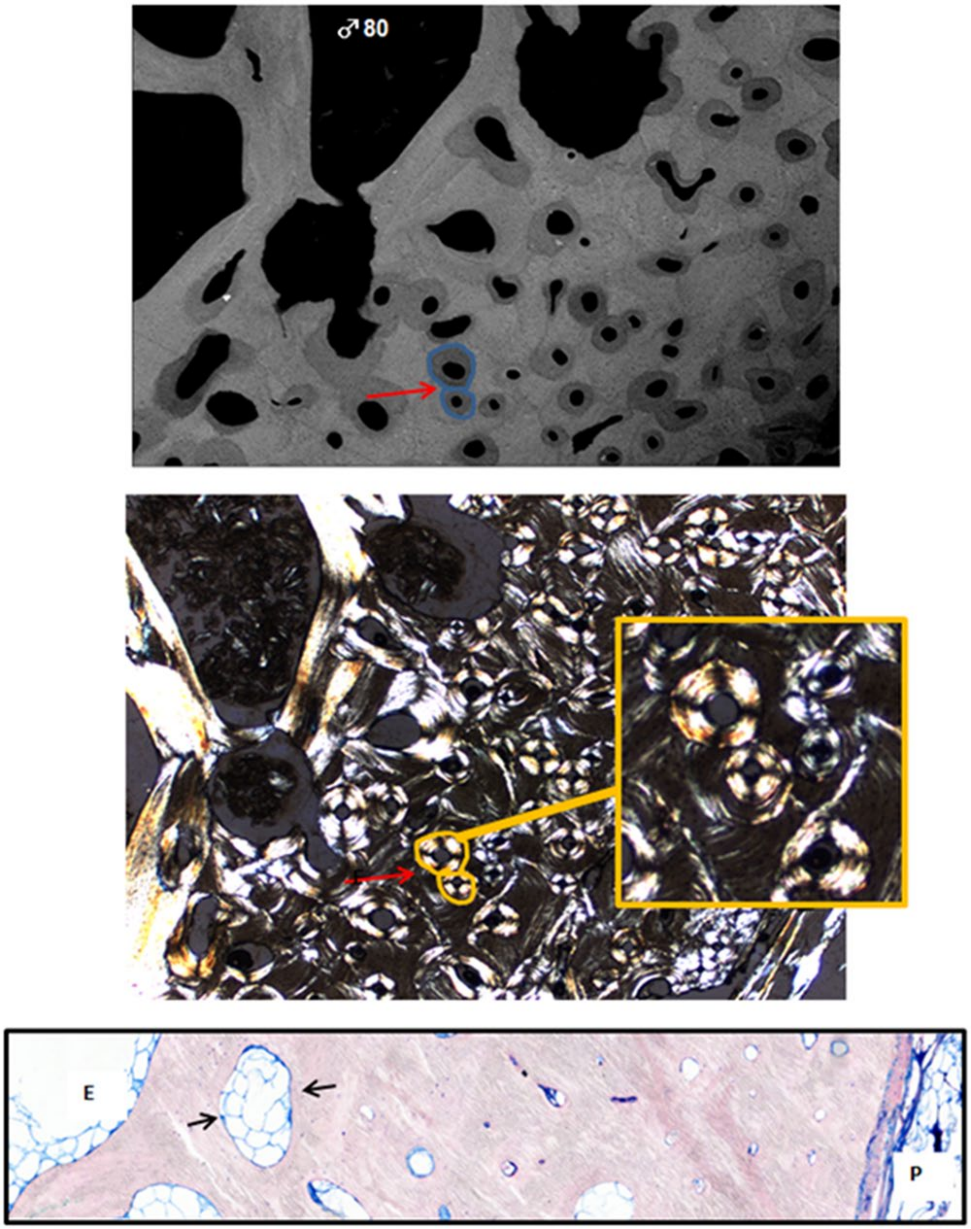
At high magnification (x2.5) from right Fig. 2, Top image shows the circular osteons present in adult bone. Note the heterogeneity of the mineralization, with dark (low mineralization) and white osteons (higher mineralization). Red arrows and blue lines point of the circular and smaller osteons. Middle image shows the corresponding section seen in polarized light. Bottom image illustrates at 8 µm thin-section of May-Grünwald Giemsa staining, from endosteal (E) to periosteal (P) area, at high magnification (x10). Black arrows shows a limited bone cellular activity, with cavity filled with giant adipocytes. Illustrations will be available at high resolution.

Figure 1 shows the evolution of the fibula with age in transverse DMB sections. Figure 2 shows a transverse fibulae section at two ages (Male 7 years old, Male 80 years old) with the specific location of the high magnification seen in Figs. 3 and 4. On juvenile bone, drifting osteons^45^ are visible and exhibit a variation in their main direction. In adult bone, those types of osteon are no more observed (Fig. 4).

**Figure 5:**
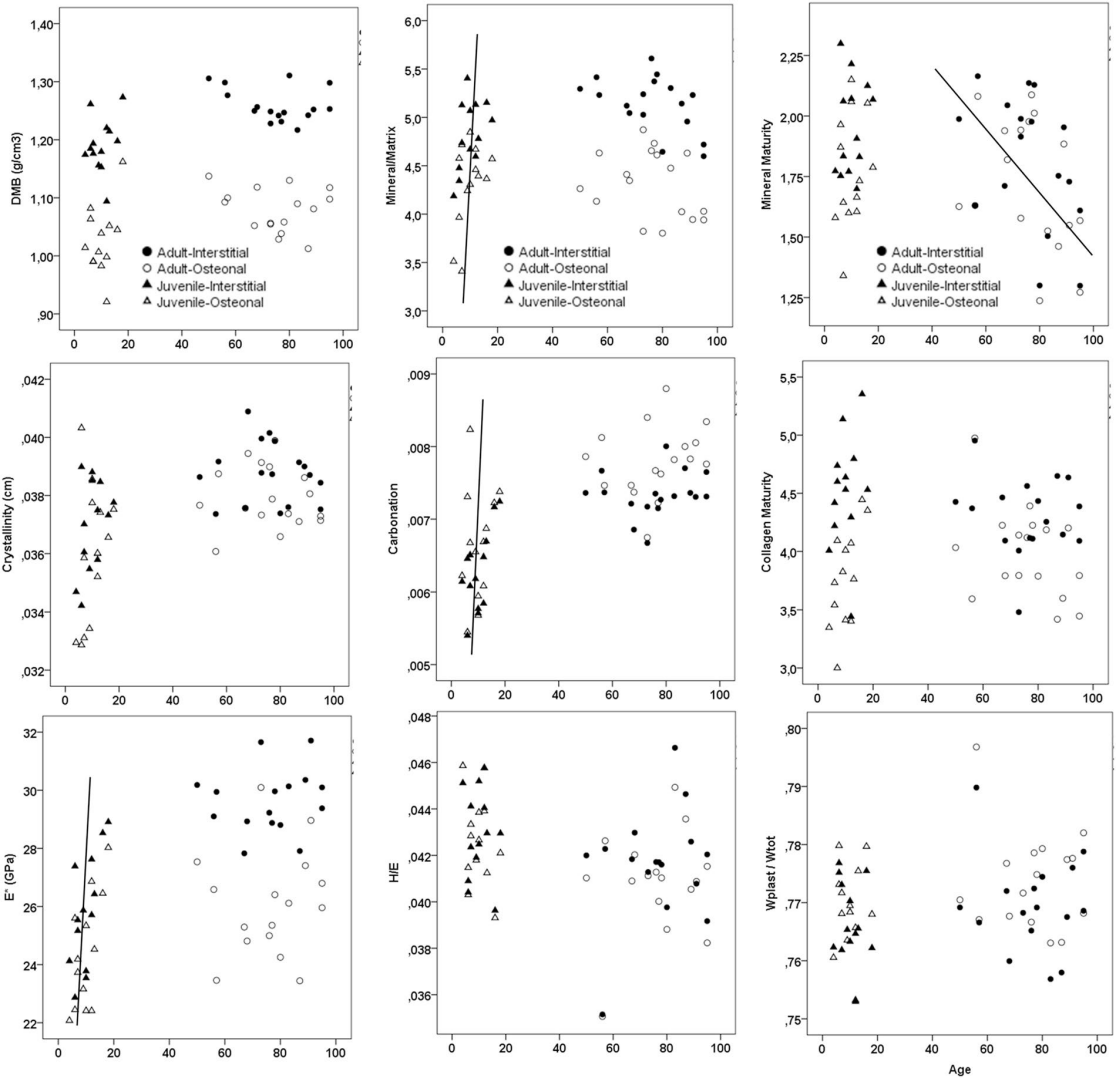
Evolution of the parameters with age for both osteonal and interstitial bone regions. Juvenile data in triangle and adult data in circle, sharp symbol for osteonal region and plane symbol for interstitial region. Solid lines represent significant interactions with age in juvenile or adult group, using the pooled osteonal and interstitial regions.

**Figure 6:**
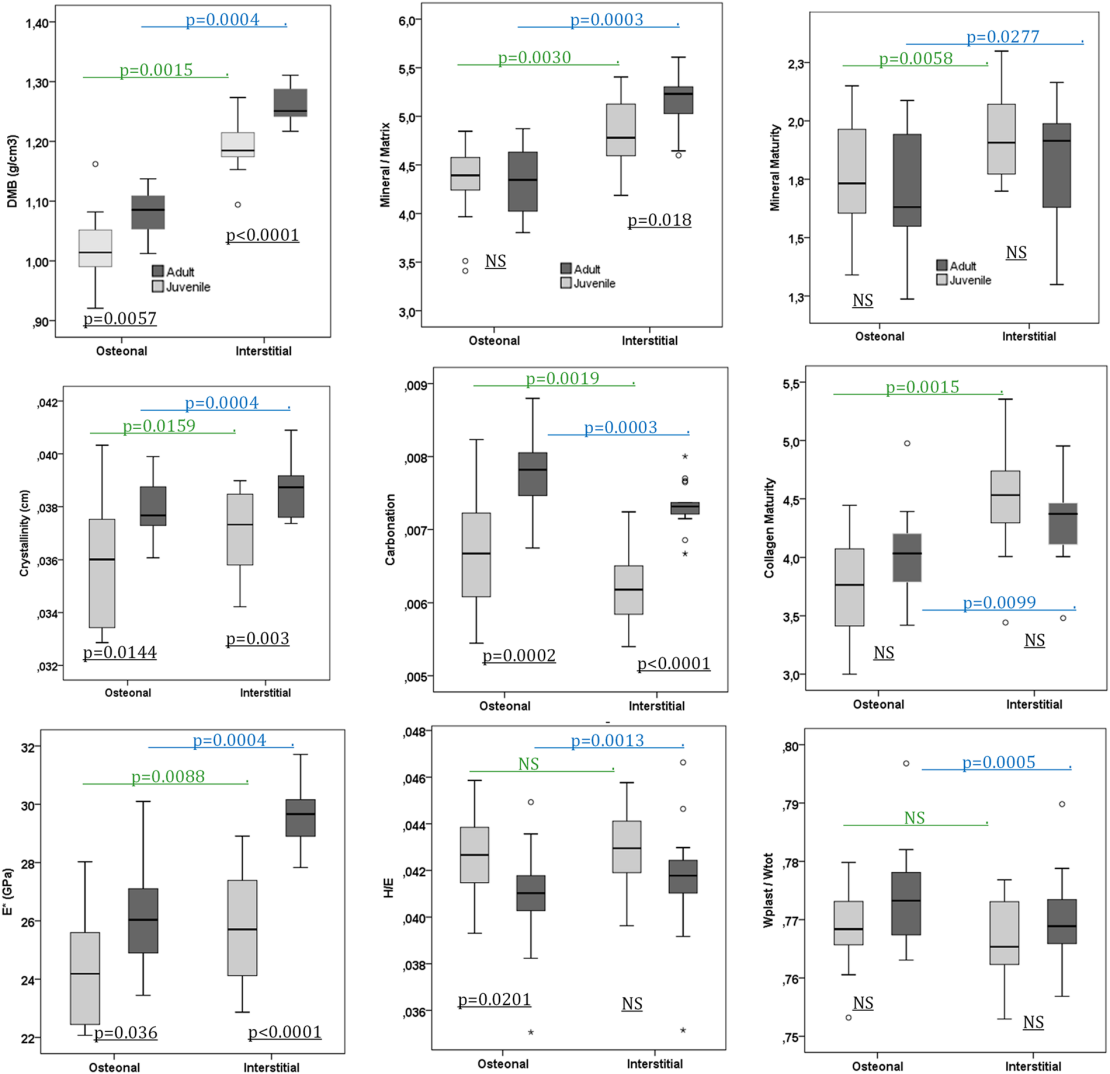
Box Plot of Juvenile data in light grey, Adults data in dark grey, for osteonal and interstitial regions. P-value comparing groups are indicated. The difference (p) between Osteonal and Interstitial is shown using a Wilcoxon paired test and the difference between Juvenile/Adult is shown using a Mann-Whitney unpaired test.

Figure 5 shows the evolution of all age-dependent variables in osteonal and interstitial areas. The gap between juveniles and adults prevents from using a linear or non-linear regression curve to evaluate the relationship between age and tissue properties. Table 1 shows the correlations between intrinsic parameters with chronological age within each subgroup (juvenile/osteonal, juvenile/interstitial, adult/osteonal, adult/interstitial). Collagen maturity and plane strain modulus (E*) were positively linked to age in the juvenile/osteonal subgroup, which was not observed in adults. E* was also positively linked to age in the juvenile/interstitial subgroup but not in adult (both areas). Mineral maturity was negatively linked to age in adult bone (both osteonal and interstitial). Compositional and indentation variables of adult bone were equivalent or higher than juvenile bone, with the exception of H/E* in osteonal tissue (p = 0.02) (Fig. 6 and Table 3).

**Table 1:** Spearman correlation coefficients (r′) obtained between chronological age and the different parameters of the FTIRM tests and the mechanical parameters obtained by indentation for the four subgroups (*p < 0.05, **p < 0.01).

Age	n	DMB	Crystallinity	Carbonation	Mineral/Matrix	Mineral Maturity	Collagen maturity	E*	H/E*	W_plast_/W_tot_
Juvenile/Osteonal	13	0.077	0.420	0.279	0.340	0.293	0.577*	0.561*	−0.185	−0.011
Juvenile/Interstitial	13	0.287	0.359	0.530	0.547	0.169	0.334	0.622*	0.072	−0.191
Adult/Osteonal	17	−0.128	−0.196	0.292	−0.299	−0.493*	−0.285	0.109	−0.026	0.075
Adult/Interstitial	17	−0.227	−0.137	0.144	−0.447	−0.492*	−0.039	0.213	0.019	0.007

### Comparison of osteonal and interstitial tissue properties in juveniles and adults

- Juvenile vs adult: (Mann-Whitney unpaired test):
  A significant difference between juveniles and adults was observed in both osteonal and interstitial areas for DMB, crystallinity, carbonation, and E* (based on the Mann-Whitney unpaired test) Fig. 6. No differences in mineral maturity, collagen maturity, and W_plast_/W_tot_ in juveniles and adults were observed. A significant difference in the mineral/matrix ratio was found in the interstitial area, whereas H/E* ratio showed a significant difference in the osteonal area.
- Osteonal vs interstitial (Wilcoxon paired test)
  Significant differences between the osteonal and interstitial areas were observed for all variables, except for the H/E* ratio and W_plast_/W_tot_ in the juvenile group.
- Interaction Category/Region: Comparison with a mixed-model (Table 3).

A mixed-model was used to evaluate the effect of the area on the interaction between juvenile and adult groups (Table 3). Differences between the juvenile and adult groups in the osteonal and interstitial regions are shown in Table 3. If the interaction is statistically significant, its sign is indicated as +/−. E* and mineral/matrix ratio showed a negative interaction which indicates that the difference between juveniles and adults is higher in the osteonal than in the interstitial area. A positive interaction was observed for collagen maturity, which indicates that the difference between adults and juveniles is higher in the interstitial area than in the osteonal area.

### Correlations between compositional and indentation tissue properties in juveniles and adults in all areas

When all data are pooled, E* correlated with all parameters (DMB, crystallinity, carbonation, mineral/matrix ratio, and collagen maturity) except for mineral maturity, whereas H/E* and W_plast_/W_tot_ correlated only with carbonation Table 2. Correlations between other parameters for each technique are shown in Table 2 Part2A.

**Table 2:** Spearman correlation coefficients (r’) obtained between the different variables of microradiography, FTIRM and indentation in: Part 2A: All samples (juvenile and adult bone) with pooled osteonal, interstitial, (*p < 0.05, **p < 0.01); Part 2B: Juvenile bone with pooled osteonal and interstitial, (*p < 0.05, **p < 0.01, BL: borderline 0.05 < p < 0.07); Part 2C: Adult bone with pooled osteonal and interstitial, (*p < 0.05, **p < 0.01).

Part 2A								
ALL (n = 30)	DMB	Crystallinity	Carbonation	Mineral/Matrix	Mineral Maturity	Collagen maturity	E*	H/E*
Crystallinity	0.375**	—						
Carbonation	0.063	0.016	—					
Mineral/Matrix	0.594**	0.499**	−0.272*	—				
Mineral Maturity	0.153	0.557**	−0.476**	0.467**	—			
Collagen maturity	0.522**	0.218	−0.052	0.555**	0.383**	—		
E*	0.710**	0.321*	0.374**	0.479**	−0.040	0.348**	—	
H/E*	−0.127	−0.057	−0.402**	0.160	0.035	−0.013	−0.257	—
W_plast_/W_tot_	0.009	−0.021	0.289*	−0.242	−0.019	−0.048	0.121	−0.893**
**Part 2B**								
**Juveniles (n** = **13)**	**DMB**	**Crystallinity**	**Carbonation**	**Mineral/Matrix**	**Mineral Maturity**	**Collagen maturity**	E*	H/E*
Crystallinity	0.326	—						
Carbonation	−0.004	−0.439*	—					
Mineral/Matrix	0.482*	0.506**	−0.185	—				
Mineral Maturity	0.471*	0.747***	−0.362	0.404*	—			
Collagen maturity	0.677***	0.343	0.067	0.644***	0.515**	—		
E*	0.573**	−0.032	0.554**	0.372^BL^	0.092	0.486*	—	
H/E*	−0.220	−0.103	−0.261	0.051	−0.273	−0.266	−0.290	
W_plast_/W_tot_	0.082	0.123	0.177	−0.192	0.316	0.136	0.154	−0.861**
**Part 2C**								
**Adults (n** = **17)**	**DMB**	**Crystallinity**	**Carbonation**	**Mineral/Matrix**	**Mineral Maturity**	**Collagen maturity**	E*	H/E*
Crystallinity	0.237							
Carbonation	−0.308	−0.664***						
Mineral/Matrix	0.561**	0.580***	−0.720***					
Mineral Maturity	0.044	0.778***	−0.620***	0.558**				
Collagen maturity	0.357*	0.221	−0.166	0.483**	0.297			
E*	0.671***	0.280	−0.420*	0.549***	0.133	0.30	—	
H/E*	0.100	0.386*	−0.298	0.326	0.171	0.283	0.087	—
W_plast_/W_tot_	−0.036	−0.434*	0.214	−0.316	−0.191	−0.235	−0.079	−0.923**

When groups are separated (Table 2. Part 2B & 2C), the link with mechanical parameter is different. Plane strain modulus is correlated with DMB and carbonation, but also with collagen maturity in juvenile bone and with mineral/matrix in adult bone. H/E* is only linked to crystallinity in the adult group. W_plast_/W_tot_ correlated with H/E* in the juvenile group, and with both H/E* and crystallinity in the adult group.

Using a multiple regression to investigate the relation between mechanical and compositional parameters (Table 4), only E* was explained by simultaneously DMB, carbonation and mineral/matrix in the juvenile group. In the adult group, plane strain modulus was only explained by DMB whereas no link was found for the juvenile group. H/E* can be explained by DMB, crystallinity, mineral maturity and collagen maturity. W_plast_/W_tot_ is explained in the model by both crystallinity and mineral maturity in the adult group.

## Discussion

The aim of this study was to challenge our hypothesis that intrinsic properties would change with age in juveniles but not in adults. Different trends were observed, depending on age and the intrinsic material properties of juvenile and adult bone. Contrary to what we hypothesized, not all the intrinsic properties of the juvenile bone changed with age compared to those of the adults. Mechanical properties were also differently explained in juvenile and adult groups.

### Chronological age in juveniles and adults in osteonal and interstitial tissue properties

Several bone properties correlated with chronological age (except for collagen maturity and W_plast_/W_tot_), including higher bone mineralization, increased crystal size/perfection and carbonation, decreased mineral maturity, and increased mechanical properties (Table 1). Based on these results, the skeleton appears to reach a stable condition in adult bone (Figs. 2(c,d), 4) compared to juvenile bone with drifting osteons (Figs. 2(a,b), 3).

E* positively correlated with age in juveniles but not in adults (Fig. 5). A study by Akkus *et al*. (2004) that analyzed rat femurs of young adult 3 months old, middle-aged 8 months old, and aged 24 months old female Sprague-Dawley rats by Raman spectroscopy demonstrated that increased mineralization, increased crystallinity, and increased type-B carbonate substitution significantly correlated with decreased elastic deformation capacity with age^46^. The results of their study demonstrated that the physicochemical status of mineral crystals of bone tissue impacts the mechanical properties of rat femoral cortical bone, as in our study.

We found in juvenile bone only, that mineral/matrix ratio, and total carbonation (types A and B and labile carbonates) increased with chronological age without changing crystallinity. This suggests that in juvenile bone mineral content and carbonation increase without change in crystal size/perfection in osteonal or interstitial bone. In adult bone, except for mineral maturity (decreasing with age), no change in mineral/matrix ratio, carbonation or crystallinity were found. Mirzaali *et al*. (2016) showed comparable results with no change in mineral content or crystallinity with donor age in the elderly^25^ when using Raman spectroscopy. Yerramshetty *et al*. (2006) also used Raman spectroscopy and showed that crystallinity does not change with age in adult human cadaveric bone (femurs, 52–85 year-old) but no distinction between tissue type was made^47^. They found a significant increase of carbonation with donor age (type B carbonates/ν1PO_4_). Different results were reported by Follet *et al*. on adult vertebral bone (54–93 year-old) where crystallinity increased with donor age and was associated with decreased carbonation (types A and B and labile carbonates)^48^. These differences may have been due to differences in bone type that were analyzed by the same technique (cortical fibula compared to trabecular vertebrae) or to differences in bone regions that were analyzed (osteonal / interstitial bone in the present study; no distinction in Follet *et al*.^48^). For comparative studies, care must then be taken to use the same type of bone and the same location, but it seems a pattern arise which is that physicochemical status and mechanical properties change with age-related.

### Comparison of osteonal and interstitial tissue properties in juveniles and adults

Material composition of bone tissue was studied at the level of osteons and interstitial tissue using microradiography and FTIRM Fig. 6. The relationship between parameters for osteonal and interstitial tissue were the same for juveniles and adults. In juveniles and adults, crystallinity, mineralization/matrix, mineral maturity, collagen maturity, DMB, and E* were lower in osteonal than in interstitial tissue, whereas carbonation and W_plast_/W_tot_ were higher. Those parameters are always higher in adult bone as compared to juvenile bone in both osteonal and interstitial tissue. Our results show that interstitial tissue had higher levels of mineralization than osteonal tissue in adult bone which is consistent with previous studies^6,18^. Our results allow to extend this feature to juvenile bone.

A study on ewes^49^ showed that during the first 6-months of mineralization, the degree of mineralization and microhardness significantly increased, followed by a slower increase until reaching maximal values at 30-months. This secondary mineralization progression was associated with an improvement in both maturation and crystal perfection of the mineral part of bone matrix^49^ which is comparable to our results. Bergot *et al*. (2009) acquired microradiographs of transverse cortical bone sections from 99 female and 94 male donors (N = 193) ranging from 20 years to 90 years of age^50^. They reported DMB for interstitial and osteonal tissue separately. Among all donors, osteonal and interstitial DMB was 88.79 ± 7.07 g/cm^3^ and 90.56 ± 7.14 g/cm^3^ with a coefficient of variation (CV) of 7.88% and 7.96%, respectively, and a percent difference ranging from 2–3.5% (p < 0.0001). In our study, CV values for osteonal and interstitial ranged from 2.30–5.65% while the mean percent difference between osteonal and interstitial tissue for the juvenile and adult groups were 5.2% and 6.9%, respectively (p < 0.0057).

FTIRM was used to study the physicochemical modifications of bone composition regarding the mineral phase as well as the organic matrix^42^. FTIRM parameters included composition of the crystals and their organization. The results from this study showed that mineral crystallinity values were lower in the juvenile group (crystals were smaller and less perfect). Additionally, significantly lower values in carbonation were observed in the juvenile group, which indicates that lower levels of CO_3_ ions were present in the core of the apatite crystal in juvenile bone. However, within each group (juvenile and adult), a different observation was done. In our study, carbonation was lower in both tissue regions in the juvenile group as compared to the adult group. Indeed, carbonation was always lower in interstitial bone than in osteonal bone. We conclude that carbonate-incorporation in bone matrix is different based on chronological age and tissue age (“young” osteon and “old” interstitial). Petra *et al*. (2005) used synchrotron infrared micro-spectroscopy to analyze femoral biopsies taken from an 11-year-old patient (femoral osteotomy), which showed that the carbonate to phosphate ratio in several osteons consistently increased (from 1%) but at a much lower intensity with increasing distance from the center of the osteon^51^. The authors concluded that their work confirmed the presence of immature forms of bone mineral (in the ν_1_ν_3_PO_4_ area phosphate groups region) in some pediatric cortical bone osteons. Unfortunately, the authors did not perform the same work with interstitial bone^51^. In our study, the variation in carbonation within the osteons was <0.1% in both juvenile and adult groups.

A study of iliac crest bone from adults without apparent signs of metabolic bone disease found a significant, but weak, correlation between mineral maturity and crystallinity^52^. The crystallinity of this previous study and our study were in the same range (interstitial bone: 0.0406 versus 0.0387 (our study); osteonal bone: 0.0397 versus 0.0379 (our study)). The same pattern was observed for mineral maturity (interstitial bone: 2.046 versus 1.813 (our study); osteonal bone: 1.738 versus 1.717 (our study)). Additionally, crystallinity and mineral maturity showed a significant correlation (p < 0.0001).

Boskey showed in an animal study that the younger animal with mechanically stronger bones has a mixture of small (recently formed) crystals and larger, more mature crystals. Indeed, the mixture of small and large crystals may represent the optimal situation for effective resistance to loads. In aging bone, the average crystal size is larger. When there are only large crystals or only small crystals present, the mechanical properties of the bone composite are considered to be weakened. Boskey’s review also points out the discrepancy between studies (e.g., Table 1 of Boskey, 2003). In a previous study of vertebrae and aging^48^, we showed an increase of both size and perfection of the crystals. Based on the previously referenced review article^29^, bone containing a greater number of large crystals becomes more brittle and tends to fracture more easily. We showed a significant correlation between crystallinity and E* but not with H/E* or W_plast_/W_tot_, which suggests that larger/more perfect crystals may not be associated with brittle bone. However, deviation from the ideal composition might be considered to be associated with the deterioration of mechanical properties at the microstructural level^53^. Indeed, stiffness of cortical bone is predominantly associated with mineral content and bone density, whereas toughness of cortical bone is strongly associated with the quality of the collagen matrix^26^. Bone’s crystalline structure (i.e., amount of crystals) provides compressive strength and brittleness; collagen fibrils provide tensile strength and toughness. Remodeling induces regional variability of collagen fiber orientation, leading to changes in bone mechanical properties. It was previously shown that the collagen network loses up to 50% of its capability to absorb energy during aging, likely because of an increase in the percentage of denatured collagen^54^, but without taking into account the porosity of bone which can account to 76% of the reduction in strength^55^.

### Correlations between compositional and indentation tissue properties in juveniles and adults within all areas

Results were different when groups were separated or pooled (only Table 2. Part.2A, see § limitations).

Yerramshetty *et al*. (2008) studied the association between mineral crystallinity and the mechanical properties of human cortical bone^30^. Using Raman scans and mechanical tests (monotonic and fatigue; n = 64) on 16 human cadaveric femurs (52–85 years old) revealed that crystallinity accounted for only 6.7% to 48.3% of the variation in monotonic mechanical properties, with a significant positive relationship between crystallinity and modulus in tension. Results of this study indicated that increased tissue-level strength and stiffness positively correlated with increased crystallinity while ductility was reduced. The authors concluded that crystallinity could be used as a complementary diagnostic marker for the prediction of bone strength. As Yerramshetty *et al*. (2008), we also found this relationship between plane strain modulus and crystallinity in juvenile or adult groups when we pool all groups, but this link disappears when groups are separated (Table 2 Part 2A, 2B, 2C). However, crystallinity was associated with H/E* and W_plast_/W_tot_ in the adults group (Table 2 Part 2C).

E* showed different correlations with compositional properties depending on the group studied (Table 2), but never correlate with H/E* or W_plast_/W_tot_. This suggests that mechanical competencies of bone are mainly acquired during childhood and growing. However, it has been shown that deterioration in bone macro-mechanical competence occurs during the aging process, mainly because of an increase in porosity. Nevertheless, porosity is not detected by microindentation and this alteration should be considered for studies on a length scale above the one used in this study^36,25,55^. Although energy absorption, fracture toughness, and ultimate tensile strain show age-related changes of approximately 5–10% per decade, elastic moduli in tension or compression degrade by only approximately 2% per decade^53,56^. However, these macroscale studies are not being representative for microscales, as toughness tests are influenced by the crack deflecting porosity. At the macroscale, changes in the mechanical competence of bone can be explained by functional adaptation of bone structure, i.e. an age-related increase in porosity and microcracks^55,57^. Each osteonal remodeling event that fails to replace all of the bone previously removed results in an increase in cortical bone Haversian porosity. Lacuno-canalicular porosity may not be detected by the indenter. The ratio of highly mineralized to new, less mineralized bone tissue is higher when bone remodeling is suppressed, which results in an increase in the homogeneity of cortical bone tissue^58^. More homogenous tissue allows cracks to grow more easily, which reduces the macroscopic toughness of the composite material^53^. However, the size of bone microcracks is an order of magnitude greater than the indentation depth and therefore will not be detected here^48,59–61^. Modifications of intrinsic mechanical properties involved in this deterioration are also an important point. Indeed, we found a positive linear correlation between age and mineral/matrix ratio and carbonation in the juvenile group but disappear in the adult group. These results reveal that those parameters were rapidly increasing during growth, but remain constant in elderly bone. DMB was highly correlated with the mechanical parameter (E*) but did not correlate with H/E* and W_plast_/W_tot_ ratio in juvenile and adult groups. Based on all data, DMB is still highly correlated to E*.

Osteonal and interstitial tissue display different mechanical behaviors in juvenile bone, but these differences are not as pronounced in adult bone. In osteonal bone, we can find primary and secondary osteons^62^. This distinction between primary (totally new) and secondary (replacement) bone is important because it is likely that control of primary bone apposition to periosteal or endosteal bone surfaces is different from that replacing preexisting bone by secondary bone^62^. Primary osteons are quite different (developmentally, morphologically and mechanically) than secondary osteons. Martin and Burr (1989) hypothesized that primary osteonal cortical bone may be mechanically stronger than secondary osteonal cortical bone^62^. Morphologically, the main distinction is that primary osteons do not have cement lines (reversal lines) because they are not the product of bone remodeling. Primary and secondary osteons are considered to be young tissue since they are the result of a remodeling cycle while interstitial tissue is considered to be old tissue. Plane strain modulus strain modulus of old tissue is higher than that of young tissue^22,40^. The first type of bone tissue that appears in embryonic development and in fracture repair (woven bone) have been recently studied in fetal/infantile bone femurs^20^. Woven bone can be distinguished from lamellar bone using a polarized light and staining (if needed)^63^. However, microindentation measurements could not distinguish between primary and secondary osteons. By the nature and definition of primary osteonal bone, we assumed that the primary osteonal bone rate is higher in juveniles and diminishes with age in adults

### Discussion of the mixed model and multiple model

E* and mineral/matrix ratio showed a negative interaction, indicating that the difference between adults and juveniles was higher in interstitial tissue as compared to osteonal tissue Tables 3 & 4. Interstitial tissue is considered mature bone tissue as compared to osteonal tissue and it is apparent that mechanical properties are higher in older and more mineralized tissue. Interaction of the H/E* and W_plast_/W_tot_ ratio was identical within osteon and interstitial bone in adults and juveniles. The interstitial tissue is considered to be “old bone” and it is known that the plane strain modulus of “old bone” is higher than that of “young bone”^22,40^.

**Table 3:** Mean and (SD, coefficient of variation %) for each parameter (osteonal and interstitial separated).

	Osteonal			Interstitial			Interaction Category/Region		
	Juvenile	Adult	p-values	Juvenile	Adult	p-values	Sense of interaction (Adult – Juvenile) (+/−)	p-values	
E*(GPa)	24.40 (1.93; 7.9%)	26.09 (1.83; 7.0%)	**0.036**	25.80 (1.93; 7.5%)	29.63 (1.11; 3.7%)	**<0.0001**	−	**<0.001**	
H/E*	0.0426 (0.0021; 4.9%)	0.0409 (0.0023; 5.6%)	0.0201	0.0429 (0.0019; 4.4%)	0.0418 (0.0026; 6.2%)	ns		0.087 (ns)	
W_plast_/ W_tot_	0.769 (0.007; 0.91%)	0.774 (0.009; 1.2%)	ns	0.767 (0.008; 1.0%)	0.767 (0.007; 0.9%)	ns		0.105 (ns)	
Crystallinity (cm)	0.0360 (0.0024; 6.7%)	0.0379 (0.0011; 2.9%)	**0.0144**	0.0370 (0.0016; 4.3%)	0.0388 (0.0011; 2.8%)	**0.003**		0.619 (ns)	
Carbonation	0.0066 (0.0008; 12.1%)	0.0078 (0.0005; 6.4%)	**0.0002**	0.0063 (0.0005; 7.9%)	0.0073 (0.0003; 4.1%)	**<0.0001**		0.51 (ns)	
Mineral/ Matrix	4.31 (0.44; 10.2%)	4.313 (0.345; 8.0%)	ns	4.819 (0.36; 7.5%)	5.141 (0.284; 5.5%)	**0.018**	−	**0.019**	
Mineral maturity	1.773 (0.235; 13.3%)	1.717 (0.271; 15.8%)	ns	1.954 (0.195; 10.0%)	1.813 (0.276; 15.2%)	ns		0.214 (ns)	
Collagen maturity	3.769 (0.422; 11.2%)	3.984 (0.391; 9.8%)	ns	4.516 (0.483; 10.7%)	4.305 (0.330; 7.7%)	ns	+	**0.016**	
DMB (g/cm^3^)	1.027 (0.058; 5.6%)	1.079 (0.037; 3.4%)	**0.0057**	1.191 (0.047; 3.9%)	1.260 (0.029; 2.3%)	**<0.0001**			0.145 (ns)

Difference Adult/Juvenile is indicated for each region, using a mixed model. The significance is also obtained (p) for each region, using a Mann-Whitney unpaired test. Interaction between Category and Region is obtained using a mixed model. The sense of the interaction (+/−) is indicated when the model is significantly relevant (p-values for interaction).

**Table 4:** Multiple regression analysis describing micromechanical variables (elastic modulus (E*), ratio (H/E*) and W_plast_/W_tot_) as a function of the degree of mineralization of bone (DMB), crystallinity, carbonation, mineral/matrix, mineral maturity and collagen maturity.

Variables		Final adjusted R²	Part correlation (β)	p-value
Dependent	Independent			
**JUVENILES GROUP**				
E*	DMB	**0.676**	**0.493**	**0.006**
	Crystallinity		−0.141	0.474
	Carbonation		**0.687**	**0.000**
	Mineral/ Matrix		**0.549**	**0.014**
	Mineral maturity		0.129	0.515
	Collagen maturity		−0.224	0.322
H/E*	DMB	0.154	0.086	0.744
	Crystallinity		−0.027	0.932
	Carbonation		−0.471	0.054
	Mineral/ Matrix		0.189	0.569
	Mineral maturity		−0.447	0.172
	Collagen maturity		−0.314	0.390
W_plast_/W_tot_	DMB	0.256	−0.103	0.678
	Crystallinity		0.143	0.631
	Carbonation		0.386	0.088
	Mineral/ Matrix		−0.580	0.073
	Mineral maturity		0.514	0.098
	Collagen maturity		0.382	0.267
**ADULTS GROUP**				
E*	DMB	**0.479**	**0.736**	**0.004**
	Crystallinity		−0.002	0.994
	Carbonation		−0.144	0.566
	Mineral/ Matrix		−0.064	0.838
	Mineral maturity		0.010	0.972
	Collagen maturity		0.036	0.829
H/E*	DMB	**0.268**	**−0.620**	**0.031**
	Crystallinity		**0.706**	**0.016**
	Carbonation		−0.361	0.229
	Mineral/ Matrix		0.285	0.441
	Mineral maturity		**−0.905**	**0.009**
	Collagen maturity		**0.433**	**0.039**
W_plast_/W_tot_	DMB	**0.330**	0.482	0.075
	Crystallinity		**−0.903**	**0.002**
	Carbonation		0.196	0.490
	Mineral/ Matrix		−0.262	0.459
	Mineral maturity		**0.884**	**0.008**
	Collagen maturity		−0.377	0.058

Multiple regressions are done separately for each category (Juvenile or Adult) with osteonal and interstitial bone pooled. Bold values indicate significant results.

Collagen maturity showed a higher difference in osteonal tissue as compared to the interstitial tissue between adults and juveniles. The organic matrix constitutes the principal toughening mechanism in bone and plays a substantial role in determining properties of energy absorption and toughness^64^. Collagen maturity cannot be attributed to a single phenomenon, since the peak of amide I is sensitive to the secondary structure of collagen, which itself can change based on mineral maturity, degree of mineralization, dehydration of collagen fibers, maturation of collagen fibers. We previously demonstrated that this ratio was not correlated to enzymatic crosslinking of collagen^65^. Increase in collagen maturity in interstitial bone is then the result of a combination of different processes (cited above).

Other parameters (e.g., crystallinity, carbonation, mineral maturity, DMB) showed a null interaction, meaning that the difference between juvenile and adult were the same for osteonal and interstitial bone.

However, using a multiple regression model, we can point out a different relationship between mechanical parameters and intrinsic properties. Whereas for the adult group all mechanical properties are linked with several intrinsic properties, in the juvenile group only the plane strain modulus is explained by simultaneously DMB, carbonation and mineral/matrix. After growth, plane strain modulus is only explained by DMB. That’s different trend is a novelty in the study of juvenile bone field.

### Limitations

In some results, we stated that no difference was observed between juvenile and adult groups. Due to the small sample size, this means that this could be due either to a lack of statistical power or to an insignificant difference. Statistical differences between osteonal and interstitial tissue of both juvenile and adult groups prevented us from pooling the tissue samples together. Indeed, when we conducted the measurements, we did not express the osteonal or interstitial region as a percentage of total bone, but instead used a fixed number of measurements per tissue type. This means that we assigned equal “weight” to those tissue types, which may not be true, especially in the case of a growing skeleton. In a recent study, Gauthier *et al*. separated those compartments and found in adult a ratio of 41% for osteonal tissue and 54.5% for interstitial tissue compare to total bone, the remain was due to osteocyte lacunae morphometric parameters, with a significant differences in shape and morphometric parameters in lacunae density, lacunae main length, and anisotropy, higher in interstitial tissue compared to osteonal one^66,67^.

In juveniles, it was difficult without polarized light to distinguish drifting osteons. Schnitzler *et al*. (2013) used histomorphometry to analyze osteons and their canals for age-related changes in numbers, size, and shape in 87 iliac crest bone samples of subjects aged 0–25 years^7^. The authors identified three types of secondary osteons: drifting, eccentric, and concentric, as previously described by Jones *et al*.^68^. The authors concluded that these structures of osteons and canals varied during growth. Large asymmetrical drifting osteons with giant active canals (remodeling space) were predominant until the mid-teens and accounted for >70% of juvenile cortical porosity. In our study, we noticed these different types of structures only in the juvenile group (as shown of Fig. 2).

Another limitation was preparation and PMMA embedding (also called infiltration) of the samples. Based on Raman spectroscopy analysis of three elderly human femurs, Nyman *et al*. found that compositional properties were still detectable in samples embedded in PMMA^69^. However, bound water is a primary contributor to the mechanical behavior of bone in that it is responsible for giving collagen the ability to confer ductility or plasticity to bone, but little is known about why bound water decreases with age in hydrated human bone, which may have had, or not, an influence on our results^25,70^. In our study, microindentation tests were conducted on PMMA infiltrated bone samples. Microindentation is particularly sensitive to sample preparation methodologies, since hydration, alcohol fixation, and inclusion and roughness of bone samples can influence test results^24,40,71–73^. According to Rodriguez Florez *et al*., who compared the effects of different preparation protocols (inclusion, i.e.: coating, resin surrounding the material or infiltration, i.e. embedding) on nanoindentation results, the E* and model viscosity values were similar for included or embedded samples^74^. However, hardness was higher for samples included in the PMMA. This seems to come from the infiltration of the resin into the pores. In this study, samples from both groups were prepared using the same protocol. Therefore, preparation methodology is not critical for making comparisons between different groups.

Another limitation concerns the samples themselves. Although our study tried to quantify parameters of growing bone, we were unable to obtain a healthy bone sample to serve as a control for the study. Our patients are the closest to a healthy patient that we could get^75^. Indeed, all samples were obtained after surgery, which meant that juvenile patients were not considered healthy at the time of sample acquisition. The ideal control bone sample would have to be obtained from a healthy individual after accidental death, for example. However, this is ethically impossible in the authors’ countries. Thus, our results should be interpreted with caution.

### Conclusions

Juvenile osteonal or interstitial bone is less mineralized, contains smaller/less-perfect apatite crystals, and is less carbonated, as compared to adult bone. Mineral and collagen maturity was not significantly different. Dominated by the mineral phase, indentation modulus and hardness were also lower in juvenile bone.

The differences between osteonal and interstitial properties are distinct in juvenile and adult bone (significant interaction term). Crystallinity, mineralization index, mineral maturity, collagen maturity, DMB, and E* were always lower in osteonal tissue than in interstitial tissue, whereas carbonation and W_plast_/W_tot_ were higher. There were no consistent trends suggesting that indicators of tissue ductility (H/E* and W_plast_/W_tot_) were different between juvenile and adult bone.

It has been clearly established that child bone has a different mechanical behavior from adult bone but, despite some recent studies on the subject, the characterization of juvenile bone remains poorly documented. The issue of the present work is to gain insight into the compositional and mechanical properties of growing cortical bone tissue and compare them to adult bone as reference material. The challenge is to improve the knowledge on juvenile bone in order to develop adapted clinical devices, based in particular on ultrasound measurements, particularly suitable for new born or children in whom anesthesia should be avoided^14,75^.

## Material and Methods

### Specimens

Bone samples were collected at the same location from the distal third of the fibula of 13 children (10 male and 3 female) 4 to 18 years old (mean age of 9.9 years ± 4.0 years) during corrective surgery for a growth plate fracture, clubfeet, or for chondrodystrophy, hypoplasia, epiphyseal dysplasia. Surgeries were performed at the Timone Hospital (Marseille, France). All children were ambulatory prior to surgery and none received medications known to affect bone remodeling. In accordance with the French Code of Public Health and after approbation of the study by the Committee for the Protection of Persons, informed consent was obtained from a legal guardian of each child. Adult bone samples were harvested from the distal third of the fibula from 17 donors (7 male and 10 female) 50 to 95 years old (mean age of 76.4 years ± 13.9 years). Autopsies were performed to build a bone sample bank (French body donation to science program, declaration number: DC-2015-2357; Laboratory of Anatomy, Faculty of Medicine Lyon Est, University of Lyon, France)

All bone samples were fixed in 70% alcohol, dehydrated in absolute alcohol, and infiltrated in methyl methacrylate (MMA), which resulted in a block of infiltrated bone samples. For quantitative microradiography, 150 μm-thick sections of the block were cut in a plane perpendicular to the Haversian canals with a precision diamond wire saw (Well, Escil, Chassieu, France). Sections were progressively ground to a thickness of 100 ± 1 μm with silicon carbide and polished with a diamond suspension (0.25 μm)^76^.

For FTIRM, sections of 2 to 5 µm were cut from the infiltrated bone samples using a Polycut E microtome (Leica, Wetzlar, Germany). The residual block of the infiltrated bone samples was used for nanoindentation testing.

All measurements detailed the biological tissue age from each region (“young” osteonal and “old” interstitial tissue) and the chronological donor age of each group (juvenile and adult).

### Quantitative microradiography

Quantitative microradiography of 100 μm-thick bone sections was performed using an X-ray diffraction unit (L9421-02, Microfocus, Hamamatsu Photonics, Japan) to assess the degree of mineralization of bone (DMB). X-ray images of bone samples and an aluminum standard were acquired with a CCD camera with the following settings: active area of 36 × 24 mm (4008 × 2671 pixels) with a 12-bit (4096 values) digital image, scintillator Gd2O2S: Tb, 12 μm aluminum filter (FDI VHR 11 M, Photonic science, Robertsbridge, UK). Each acquisition was an average of five images with an exposure time of 7-seconds per image. Due to the high magnification, multiple areas were needed to rebuild an entire sample. After calibration of gray level using the aluminum standard^76^, the mean gray level of BSUs was converted into degree of mineralization values (in g/cm^3^). For each bone sample, 20 BSUs (10 osteons and 10 interstitial areas) were individually selected by drawing a region-of-interest (ROI) around the BSU and analyzed to obtain DMB values for these areas by a unique operator. The entire bone section was also analyzed to obtain an average DMB value^41^.

### Fourier-transform infrared microspectroscopy (FTIRM)

A GXII Auto-image microscope (Perkin-Elmer, Norwalk, CT, USA) equipped with a wideband detector (mercury–cadmium–telluride) (7800–400 cm^−1^) was used to perform FTIRM in transmission mode on 2 μm-thick sections. A Cassegrain objective (numerical aperture of 0.6) with a spatial resolution of 10 μm at typical mid-infrared wavelengths (4000–400 cm^−1^) was used for measurements. Osteonal and interstitial bone were clearly identified under the device’s microscope. Twenty measurements per sample (10 measurements in osteonal and interstitial regions) with a spatial resolution of 40 × 40 μm were performed. Each spectrum was collected at a resolution of 4 cm^−1^ and 50 scans per spectrum. Contributions to the spectrum of air and MMA were subtracted from the original spectrum. Following automatic baseline correction (Spectrum Software) and curve fitting of every individual spectrum, bone characteristics were quantified using GRAMS/AI software (Thermo Galactic, Salem, NH, USA)^77,78^.

In a bone spectrum (Fig. 1), six distinct regions were identified based on the vibrational response of different constituents:

- The amide (I, II, III) areas [1300, 1700] cm^−1^, corresponding to the signal of the organic matrix in the bone tissue.
- The ν_1_ν_3_PO_4_ area [900, 1,200] cm^−1^, corresponding to symmetric and antisymmetric stretching of the phosphates.
- The ν_2_CO_3_ area [800, 900] cm^−1^, corresponding to symmetrical stretching of the carbonates.
- The ν_4_PO_4_ area [500, 650] cm^−1^, corresponding to antisymmetric deformation of the phosphates.

The following five variables within these regions were determined to characterize each sample:

Crystallinity, which is calculated as the inverse of the full-width at half-maximum (1/FWMH) parameter of the 604 cm^−1^ peak (apatitic phosphate environment) that corresponds to both crystal size and perfection^52^.

Ratio of mineral to organic matrix, (mineral/matrix) which is the area ratio of the [910–1184] cm^−1^/[1592–1730] cm^−1^ bands describing mineral over organic matrix ratio^43^;

Mineral maturity, which is the area ratio of the apatitic phosphate over non-apatitic phosphate (1030/1110 cm^−1^ area ratio) that reflects the age of mineral^52^.

Collagen maturity, which is the ratio of organic matrix bands (1660/1690 cm^−1^ area ratio) that reflects the change in secondary structure of collagen in relation to the mineralization process^43,65^.

Carbonation, which is the ratio of the ν_2_CO_3_ area [862, 894] cm^−1^ to the ν_1_ν_3_PO_4_ area [910, 1184] cm^−1^ that reflects the incorporation of CO_3_ ions into the crystal.

### Microindentation tests

Flat and parallel surfaces on the residual blocks of infiltrated samples were produced with an ultra-miller (Polycut E, Reichert-Jung, Germany). Indentations were performed under dry conditions with an Ultra Nano Hardness Tester (UNHT, CSM Instruments, Switzerland) equipped with a long-shaft reference tip and a Berkovich indenter. Five indentations were made on 10 osteonal and 10 interstitial regions in a plane perpendicular to the bone axis.

To minimize the effect of creep on the measurements, a trapezoidal protocol in load control up to a maximum depth of 1 µm with a loading rate of 100 mN/min, a holding time at maximum force of 30 seconds^33^, and an unloading rate of 400 mN/min was used^25^. Plane strain modulus (E^*^, GPa), indentation hardness (H, GPa), elastic work (W_elast_), and total work (W_tot_) were extracted^25^. Plane strain modulus was recovered from the experimentally measured reduced modulus E_r_^79^ for known isotropic constants E_i_ and ν_i_ of the diamond indenter tip using Eq. (1). Parameters were measured on the unload curves.

1 $$E={(\frac{1}{{E}_{r}}-\frac{1-{v}_{i}^{2}}{{E}_{i}})}^{-1}$$

Indentation hardness is given by the ratio of maximum load (P_max_) to contact area at maximum depth A_c_

2 $$H=\frac{{P}_{max}}{{A}_{c}}$$

The total, elastic, and plastic works are defined as:

3 $${W}_{tot}={\int }_{0}^{{h}_{m}}Pdh,{W}_{elast}={\int }_{{h}_{m}}^{{h}_{p}}Pdh,{W}_{plast}={W}_{tot}-{W}_{elast}$$

with load P, depth h, maximum depth h_m_, and residual depth h_p_. For analyses, the ratios W_plast_/W_tot_ and H/E* were used since they represent surrogate measurements of ductility and yield strain, respectively^25^.

### Statistical analysis

Statistical analysis was performed in SPSS 20.0 (IBM, Amonk, NY, USA) using a significance level of 5%. Depending on the main parameters (E*, DMB, carbonation and crystallinity), the power analysis range from 68% to 99% in osteonal tissue and from 95% to 99% in interstitial tissue. All tests were two-tailed. Results are reported as scattergram and boxplots. Distribution of variables was tested with the Shapiro–Wilk procedure. Non-parametric tests were used to evaluate variables that were not normally distributed. The influence of the microstructure on mechanical behavior was studied using bivariate correlations that were tested by the Spearman’s rank correlation test. The Mann-Whitney unpaired test was used to test for differences between juvenile and adult groups (category). The Wilcoxon paired test was used to test for differences between osteonal and interstitial tissue (region) for each category.

Due to the study design, a measurement of heterogeneity based on standard deviation of the parameters was used. Those results show that the heterogeneity is the same between juvenile and adult groups in osteonal and interstitial bone for all parameters obtained with the FTIRM and microradiography technics. In indentation, heterogeneity is different between juvenile and adult groups in osteonal and interstitial bone for W_plast_/W_Tot_ and only in interstitial bone for H/E*.

A mixed model is used in cases of fixed and random effects. Our custom mixed-model evaluates the interaction between category and region, using R statistics package. The function used in this analysis, lme, is a generic function that fits a linear mixed-effects model in the formulation described by Laird and Ware (1982) but allows for nested random effects^80^. Within-group errors are allowed to be correlated and/or have unequal variances. Significance (p) is also obtained for each region. The sign of the interaction (+/−) is indicated when the model is significant (based on p-values). The interaction is positive when the difference between adults and juveniles is higher in the osteonal area than in the interstitial area. A negative interaction indicates that the difference between adults and juveniles is higher in the interstitial area than in the osteonal area. A non-significant interaction indicates that the equivalent differences are observed between adults and juveniles within the two tissue areas.

Within each group, a multiple regression was used to explain the variations of the dependent variables (mechanical properties) by the variations of the independent ones (compositional properties). Adjusted regression coefficients R² are indicated in bold if significance was found.
